# On the existence of collective interactions reinforcing the metal-ligand bond in organometallic compounds

**DOI:** 10.1038/s41467-023-39498-y

**Published:** 2023-07-03

**Authors:** Jordi Poater, Pascal Vermeeren, Trevor A. Hamlin, F. Matthias Bickelhaupt, Miquel Solà

**Affiliations:** 1 Departament de Química Inorgànica i Orgànica & Institut de Química Teòrica i Computacional (IQTCUB), Universitat de Barcelona, Martí i Franquès 1-11, 08028 Barcelona, Spain; 2 ICREA, Pg. Lluís Companys 23, 08010 Barcelona, Spain; 3 Department of Chemistry and Pharmaceutical Sciences, Amsterdam Institute of Molecular and Life Sciences (AIMMS), Vrije Universiteit Amsterdam, De Boelelaan 1083, 1081 HV Amsterdam, The Netherlands; 4 Institute of Molecules and Materials (IMM), Radboud University, Heyendaalseweg 135, 6525 AJ Nijmegen, The Netherlands; 5 Department of Chemical Sciences, University of Johannesburg, Auckland Park, Johannesburg, 2006 South Africa; 6 Institut de Química Computacional i Catàlisi (IQCC) and Departament de Química, Universitat de Girona, C/ M. Aurèlia Capmany, 69, 17003 Girona, Catalonia Spain

**Keywords:** Quantum chemistry, Computational chemistry

**arising from** S. Sowlati-Hashjin et al*. Nature Communications* 10.1038/s41467-022-29504-0 (2022)

Recently, Sowlati-Hashjin et al.^1^ concluded that the nature of the Li–C chemical bond in LiCF_3_ differs significantly from that in LiCPh_3_ (Ph = phenyl). Whereas the Li–C bond of LiCF_3_ is classified as a conventional two-center two-electron bond (exchange-correlation interaction collectivity index, ICI_XC_ = 0.910, ICI_XC_ > 0.9 and close to 1), that of LiCPh_3_ is categorized as a collective bond (ICI_XC_ = 0.393). The authors claim that collective bonds take place in systems composed of MAR_3_ (M = metal; A = C, B or Al; R = substituent) when M forms a stronger bond with the substituents R than with the central atom A. They claim the M–A interaction is either destabilizing or weakly stabilizing, whilst the 1,3-M•••R interactions are strongly stabilizing, but their method does not provide a causal mechanism that would demonstrate the correctness of this interpretation of the ICI_XC_ index. Here, we prove the opposite, namely, that the Li–CPh_3_ bond is not reinforced or provided by collective interactions, but that it is weakened by 1,3-M•••R contacts, which reduce the bond overlap. On top of that, there is 1,3-M•••R closed-shell overlap that further reduces the stability through Pauli repulsion. Taken together, our results suggest that there is no need to define the collective interaction as a new type of chemical bond.

We analyze the Li–C bond in LiCR_3_ (R = F, Ph) using quantitative Kohn-Sham molecular orbital (MO) theory in conjunction with the activation strain model (ASM) and a matching energy decomposition analysis (EDA)^2–4^ at M06-2X/TZ2P^5^ with the Amsterdam Density Functional (ADF)^6,7^ program. We stress that our physical model that provides causal relationships and thus explanations is the MO model and not, as often incorrectly stated, EDA. The latter is a tool that quantifies features in the MO bonding mechanism. Additionally, we analyze the Li–C bond in the doublet ground state of LiCR_2_^•^ and the triplet ground state of LiCR^••^ using the optimized geometry of the parent molecule (Supplementary Fig. 1).

Table 1 gathers the results of the ASM and EDA for the homolytic Li–CR_n_ (R = F, Ph; *n* = 1–3) bond cleavage into Li· + ·CR_n_ radicals^8,9^. The ∆*E*_oi_ component is the most important stabilizing contribution in the homolytic dissociation of LiCF_3_, which is driven by the bonding overlap S and energy difference ∆*ε* between the SOMOs of Li· and ·CF_3_ (Supplementary Figs. 2 and 3). In addition, there is only a weak donor–acceptor interaction between the lone pair on the F atoms and the SOMO and LUMO of Li (Supplementary Fig. 4). Thus, the Li–C bond behaves as a typical electron-pair bond between Li· and ·CF_3_, and hence, is a polar covalent interaction. Interestingly, the Li–C bond becomes stronger going from LiCF_3_ to LiCF_2_^•^ to LiCF^••^, due to stabilization of the SOMO(·CF_n_) because reducing the number of F substituents reduces antibonding overlap between the C 2*p*_z_ and F 2*p*_z_ atomic orbitals (Supplementary Figs. 2 and 3). At difference, Δ*V*_elstat_ slightly decreases from LiCF_3_ to LiCF^••^, by only around 3 kcal mol^–1^.

**Table 1:** Homolytic activation strain and energy decomposition analyses (in kcal mol^–1^) of LiCR_n_ (R = F, Ph; *n* = 1–3), singly-occupied molecular orbitals (SOMOs) energy difference (∆*ε* in eV) and overlap integrals.^a^

Species	∆*E*	∆*E*_strain_	∆*E*_int_	∆*E*_Pauli_	∆*V*_elstat_	∆*E*_oi_	∆*ε*	Overlap^b^
**LiCF**_**3**_	–63.6	15.1	–78.6	46.6	–29.7	–95.5	4.8	0.317
**LiCF**_**2**_**·**	–83.9	10.6	–94.6	35.1	–28.1	–101.6	6.3	0.322
**LiCF··**	–122.2	1.6	–123.8	21.8	–27.0	–118.6	8.7	0.330
**LiCPh**_**3**_	–45.5	4.9	–50.4	157.1	–109.3	–98.2	1.1	0.071
**LiCPh**_**2**_**·**	–43.0	9.3	–52.3	114.0	–83.2	–83.2	2.5	0.167
**LiCPh··**	–84.5	1.9	–86.4	68.7	–56.6	–98.5	5.1	0.229

^a^Computed at M06-2X/TZ2P. d(Li–C) = 1.997 and 1.980 Å for LiCF_3_ and LiCPh_3_, respectively. LiCF^••^ and LiCPh^••^ are in the triplet state, which corresponds to their valence state in the full parent molecule.

^b^Overlap integrals < SOMO(Li·) | SOMO(·CPh_x_) > forming the Li–C pair bond. See also Supplementary Table 1 and Supplementary Figs. 13–16.

Moving to LiCPh_3_, we find that the Li–C bond dissociation energy is around 20 kcal mol^–1^ less stabilizing than for LiCF_3_ (Table 1). This weakening originates from the more destabilizing Pauli repulsion, due to the larger steric size and slightly shorter Li–R distance of R = Ph compared to R = F. For the same reason, Δ*V*_elstat_ becomes more stabilizing, leading to a Li–C in the LiCPh_3_ system with a nearly balanced ratio between the electrostatic (Δ*V*_elstat_) and covalent (Δ*E*_oi_) contributions. The Δ*E*_oi_, on the other hand, is similar to that of LiCF_3_; whereas LiCPh_3_ shows a weaker electron-pair bonding than LiCF_3_, this is compensated by stronger donor–acceptor interactions (Supplementary Table 4 and Supplementary Figs. 5–8).

These data indicate that the nature of the Li–C bond is similar in LiCF_3_ and LiCPh_3_ and do not favor a classification of the Li–C bond as conventional in LiCF_3_ *versus* collective in LiCPh_3_. Additionally, if we had a collective bond in LiCPh_3_, we should expect a reduction of the Li–C bond strength when going from LiCPh_3_ to LiCPh_2_^•^ to LiCPh^••^. We, in fact, see the exact opposite, namely, the Li–C bond strength increases when going from LiCPh_3_ to LiCPh^••^, mainly because of the reduced destabilizing Pauli repulsion due to the steric repulsion of the Ph groups, while the contribution of ∆*E*_oi_ to the Li–C interaction remains more or less constant from LiCPh_3_ to LiCPh^••^.

Notably, the in-phase overlap (Fig. 1, in purple) between the SOMO of Li (2*s*) and the SOMO of CR_3_ (2*p*_z_ fragments) to form the Li–C bond is much larger for LiCF_3_ than for LiCPh_3_. At difference, the out-of-phase overlap (in orange) with the R groups is larger for the latter. Thus, the weaker Li–C interaction in LiCPh_3_, despite the expected collective interactions that should make it stronger, is in part also due to the cancelation of bond overlap that reduces the bond strength instead of providing bonding. The addition of more substituents in contact with Li drives to the cancellation of bond overlap as evidenced by the decrease in overlap densities from LiCPh_3_ to LiCPh_2_^•^ to LiCPh^••^ (Supplementary Fig. 12). In other words, the collectivity of contacts reduces the bond strength instead of generating extra stability. This cancellation effect can also be observed in the comparison between LiCF_3_ discussed above to *i*-LiCF_3_, which has also been considered to possess collective interactions (Supplementary Fig. 11). Thus, the collectivity of contacts reduces the bond strength and hence affords no additional stability. This latter statement is also supported by the computed EDA-NOCV deformation densities (Supplementary Fig. 18), which indicate similar orbital interactions in LiCF_3_, *i*-LiCF_3_, and LiCPh_3_.

**Fig. 1:**
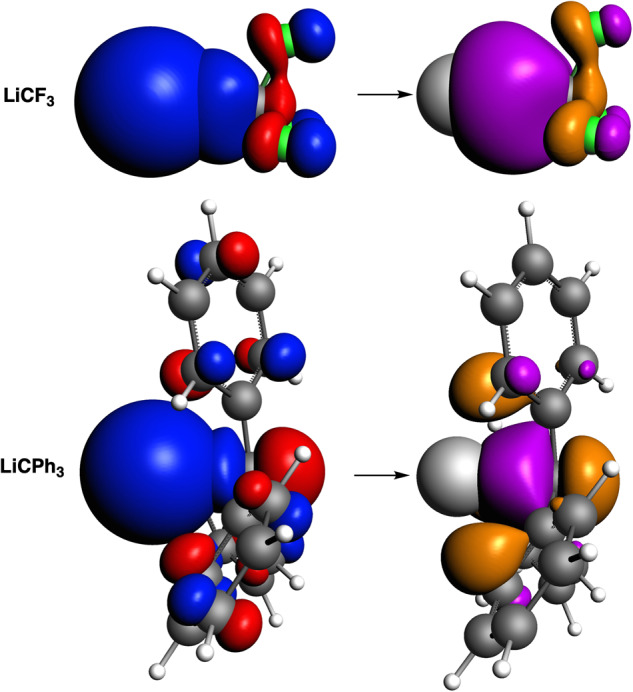
Overlap density between the two singly-occupied molecular orbitals (SOMOs) of LiCF_3_ and LiCPh_3_. Isosurfaces of the superposed SOMO of Li• and the SOMO of •CR_3_ (R = F, Ph) to construct LiCF_3_ and LiCPh_3_ (left, isovalue = 0.05 a.u.). Red and blue isosurfaces represent positive and negative phases. Overlap density between the two SOMOs (right, isovalue = 0.001 a.u.). Purple and orange isosurfaces indicate in-phase and out-of-phase overlap, respectively. See also Supplementary Figs. 9–12.

In conclusion, our quantitative MO and EDA study does not reveal any sign of collective interaction in LiCPh_3_ that makes the Li–C interaction stronger, but, in fact, the opposite. The small ICI_XC_, calculated as V_XC_(Li–C)/V_XC_(Li–{T}) where T stands for the set of all atoms of the system except the C directly attached to Li, of LiCPh_3_ of Sowlati-Hashjin et al.^1^ is likely the result of dividing a relatively low V_XC_(Li–C), because of the high polarity of this bond, by a large number of small long-range V_XC_(Li–C_Ph_) and V_XC_(Li–H_Ph_) contributions. It does not reflect any special chemical bond in this species, let alone a strong through-space interaction between the Li and the phenyl groups that could indicate collective bonding. Our results show that the nature of the Li–C bond of LiCPh_3_ does not differ significantly from that of LiCF_3_, but only that the former has weaker electron-pair bonding, which is compensated by stronger donor–acceptor interactions. Finally, we have analyzed the inverted LiCF_3_ cluster. Not unexpectedly, in this case, the ICI_XC_ is small, but this is simply the result of a low V_XC_(Li–C) because of the large distance between Li and C and a large V_XC_(Li–F) due to the short Li–F distance (Supplementary Fig. 1 and Supplementary Table 2). Lastly, we anyway do not see a need to rebrand collective bonding as a *new* flavor of the chemical bond. The concept has been invoked already in other cases and is therefore not novel^10,11^.

## Methods

All calculations were performed with the Amsterdam Density Functional (ADF) module of the AMS2021 software package at the M06-2X/TZ2P level of theory^5–7,12^. The geometry optimizations were carried out without symmetry constraints (Supplementary Table 5). Numerical Hessians were computed to characterize the optimized structures as minima (zero imaginary frequencies). Geometries for LiCX_2_• and LiCX•• (X = F or Ph) were not allowed to relax but performed at the same geometry of LiCX_3_ with the removal of either one (doublet state) or two X substituents (triplet state), respectively. LiCX_2_• and LiCX•• were computed at their doublet or triplet state, respectively. For this latter, the singlet state has been proven to be higher in energy in all cases.

The Li–C interaction was analyzed within the framework of quantitative Kohn-Sham molecular orbital theory in combination with a quantitative activation strain model (ASM) and energy decomposition analysis (EDA) in the gas phase. Both homolytic and heterolytic breaking schemes of Li–C bond have been studied. For instance, in LiCF_3_, we may have Li• (one unpaired alpha electron) and •CF_3_ (one unpaired beta electron) fragments for the homolytic breaking. Or we may have Li^+^ and ^–^CF_3_ fragments in case of the heterolytic breaking.

### Activation strain and energy decomposition analysis

For the activation strain model (ASM), the bond energy ∆*E* between two fragments is made up of two components^3,13^:

1 $$\triangle E={\triangle E}_{{{{{{\rm{strain}}}}}}}+{\triangle E}_{{{{{{\rm{int}}}}}}}$$

Here, the strain energy ∆*E*_strain_ is the amount of energy required to deform the fragments from their equilibrium structure to the geometry that they acquire in the overall complex. The interaction energy ∆*E*_int_ corresponds to the actual energy change when the geometrically deformed fragments are combined to form the overall complex.

We further analyze the interaction ∆*E*_int_ in the framework of the Kohn-Sham molecular orbital (MO) model, by dissecting it through our canonical energy decomposition analyses (EDA) into the electrostatic attraction, the Pauli repulsion and the (attractive) orbital interactions:

2 $${\triangle E}_{{{{{{\rm{int}}}}}}}={\triangle V}_{{{{{{\rm{elstat}}}}}}}+{\triangle E}_{{{{{{\rm{Pauli}}}}}}}+{\triangle E}_{{{{{{\rm{oi}}}}}}}$$

The term ∆*V*_elstat_ corresponds to the classical electrostatic interaction between the unperturbed charge distributions of the fragments in the geometry they possess in the complex. This term is usually attractive. The Pauli repulsion ∆*E*_Pauli_ between these fragments comprises the destabilizing interactions, associated with the Pauli principle for fermions, between occupied orbitals and is responsible for the steric repulsion. The orbital interaction ∆*E*_oi_ between these fragments in any MO model, and therefore also in Kohn-Sham theory, accounts for electron-pair bonding (the SOMO–SOMO interaction), charge transfer (empty/occupied orbital mixing between different fragments), and polarization (empty/occupied orbital mixing on one fragment due to the presence of another fragment). The orbital interaction energy ∆*E*_oi_ can be further decomposed into the contributions from each irreducible representation Γ of the interacting system. The use of M06-2X gives a term that cannot be decomposed, which is a correction term, such that the total orbital interaction is the correct one.

### Voronoi deformation density (VDD) charge

The electron density distribution is analyzed by using the Voronoi deformation density (VDD) method for atomic charges^14^. The VDD atomic charge ${Q}_{{{{{\rm{A}}}}}}^{{{{{\rm{VDD}}}}}}$ is computed as the (numerical) integral of the deformation density Δ*ρ*(**r**) = *ρ*(**r**) – ∑_B_ *ρ*_B_(**r**) in the volume of the Voronoi cell of atom A [Eq. (3)]. The Voronoi cell of atom A is defined as the compartment of space bound by the bond midplanes on and perpendicular to all bond axes between nucleus A and its neighboring nuclei (cf. the Wigner-Seitz cells in crystals)^14^.

3 $${Q}_{A}^{{{{{{\mathrm{VDD}}}}}}}=-\int_{{{{{{\rm{Voronoi}}}}}}\,{{{{{\rm{cell}}}}}}\,{{{{{\rm{of}}}}}}\,{{{{{\rm{A}}}}}}}\left[\rho \left({{\bf{r}}}\right)-\mathop{\sum}\limits_{B}{\rho }_{B}\left({{\bf{r}}}\right)\right]d{{\bf{r}}}$$

In Eq. (3), *ρ*(**r**) is the electron density of the molecule and ${\sum }_{{{{{\rm{B}}}}}}{\rho }_{{{{{\rm{B}}}}}}({{{{{\bf{r}}}}}})$ the superposition of atomic densities *ρ*_B_ of a fictitious promolecule without chemical interactions that is associated with the situation in which all atoms are neutral. The interpretation of the VDD charge ${Q}_{{{{{\rm{A}}}}}}^{{{{{\rm{VDD}}}}}}$ is rather straightforward and transparent. Instead of measuring the amount of charge associated with a particular atom A, ${Q}_{{{{{\rm{A}}}}}}^{{{{{\rm{VDD}}}}}}$ directly monitors how much charge flows, due to chemical interactions, out of (${Q}_{{{{{\rm{A}}}}}}^{{{{{\rm{VDD}}}}}}\, > \,0$) or into (${Q}_{{{{{\rm{A}}}}}}^{{{{{\rm{VDD}}}}}}\, < \,0$) the Voronoi cell of atom A, that is, the region of space that is closer to nucleus A than to any other nucleus.

### ETS-NOCV calculations

The natural orbitals for chemical valence method (ETS-NOCV) have been computed at the same M06-2X/TZ2P level of theory. It allows one to visualize the alteration in the electronic structure of the interacting species, which is associated with bond formation. In particular, we have depicted the deformation densities, whose shapes provide a visualization of the associated pairwise orbital interactions^15^.

## Supplementary information

Supplementary Information

## Data Availability

All data generated or analyzed during this study are included in this published article (and its supplementary information files).
