# Supplementary material for: On the existence of collective interactions reinforcing the metal-ligand bond in organometallic compounds
Source: Nat Commun. 2023 Jul 3;14:3872. doi: 10.1038/s41467-023-39498-y (PMC10318040; doi:10.1038/s41467-023-39498-y)

SUPPLEMENTARY INFORMATION

On the existence of collective interactions reinforcing the metal-ligand bond in organometallic compounds

Jordi Poater[1,2], Pascal Vermeeren[3], Trevor A. Hamlin[3], F. Matthias Bickelhaupt[3,4,5]* & Miquel Solà[6]*

[1] Departament de Química Inorgànica i Orgànica & Institut de Química Teòrica i Computacional (IQTCUB), Universitat de Barcelona, Martí i Franquès 1-11, 08028 Barcelona, Spain.
[2] ICREA, Pg. Lluís Companys 23, 08010 Barcelona, Spain.
[3] Department of Chemistry and Pharmaceutical Sciences, Amsterdam Institute of Molecular and Life Sciences (AIMMS), Vrije Universiteit Amsterdam, De Boelelaan 1083, 1081 HV Amsterdam, The Netherlands.
[4] Institute of Molecules and Materials (IMM), Radboud University, Heyendaalseweg 135, 6525 AJ Nijmegen, The Netherlands.
[5] Department of Chemical Sciences, University of Johannesburg, Auckland Park, Johannesburg 2006, South Africa.
[6] Institut de Química Computacional i Catàlisi (IQCC) and Departament de Química, Universitat de Girona, C/ M. Aurèlia Capmany, 69, 17003 Girona, Catalonia, Spain.

SUPPLEMENTARY TABLES

Supplementary Table 1. Homolytic and heterolytic activation strain and energy decomposition analyses (in kcal mol^{-1}) of $LiCR_n$ (R = F, Ph; n = 1–3), Voronoi deformation density (VDD) charges (in a.u.) of the Li fragment, and overlap integrals.[a]

Species	fragments	ΔE	ΔE_{strain}	ΔE_{int}	ΔE_{Pauli}	ΔV_{elstat}[b]	ΔE_{oi}[b]	overlap[c]	Q_{Li}
**$LiCF_3$**	Li· / ·CF_3	–63.6	15.1	–78.6	46.6	–29.7	–95.5	0.317	0.495
						(24%)	(76%)		
	Li^+ / $^-CF_3$	–150.0	2.9	–152.9	34.9	–167.9	–19.9		
						(89%)	(11%)		
**$LiCF_2$·**	Li· / ·CF_2·	–83.9	10.6	–94.6	35.1	–28.1	–101.6	0.322	0.492
						(22%)	(78%)		
	Li^+ / $^-CF_2$·	–152.7	1.8	–154.5	28.0	–159.7	–22.8		
						(88%)	(12%)		
**LiCF:**	Li· / ·CF:	–122.2	1.6	–123.8	21.8	–27.0	–118.6	0.330	0.481
						(19%)	(81%)		
	Li^+ / $^-$CF:	–156.9	0.8	–157.8	25.1	–159.0	–23.9		
						(87%)	(13%)		
**$LiCPh_3$**	Li· / ·CPh_3	–45.5	4.9	–50.4	157.1	–109.3	–98.2	0.071	0.340
						(53%)	(47%)		
	Li^+ / $^-CPh_3$	–135.6	4.1	–139.7	18.9	–106.2	–52.4		
						(67%)	(33%)		
**$LiCPh_2$·**	Li· / ·CPh_2·	–43.0	9.3	–52.3	114.0	–83.2	–83.2	0.167	0.393
						(50%)	(50%)		
	Li^+ / $^-CPh_2$·	–138.4	3.1	–141.5	17.1	–111.2	–47.4		
						(70%)	(30%)		
**LiCPh:**	Li· / ·CPh:	–84.5	1.9	–86.4	68.7	–56.6	–98.5	0.229	0.446
						(37%)	(63%)		
	Li^+ / $^-$CPh:	–146.5	1.6	–148.0	19.9	–130.0	–37.9		
						(77%)	(23%)		

[a] Computed at M06-2X/TZ2P. d(Li–C) = 1.997 and 1.980 Å for $LiCF_3$ and $LiCPh_3$, respectively. For LiCF: and LiCPh:, we have considered the triplet state, which is lower in energy than the singlet by 23.4 and 16.9 kcal mol^{-1}, respectively. [b] The values in parentheses are the percentage contributions to the total attractive interactions (ΔV_{elstat} + ΔE_{oi}). [c] Overlap integrals <SOMO(Li·) | SOMO(·CPh_x)> forming the Li–C pair bond. SOMO stands for Singly-Occupied Molecular Orbital.

As one goes from homolytic to heterolytic bond formation, both $LiCF_3$ and $LiCPh_3$ show a weakening of ΔE_{oi} and an even more pronounced strengthening in ΔV_{elstat}, leading to a weaker bond energy for the former than for the latter. The physical mechanisms behind these trends have previously been investigated for the parent compound $LiCH_3$:[9] (1) the orbital interactions in the homolytic bond formation channel benefit from an extra contribution to the stabilization, stemming from the electron in the SOMO of more electropositive lithium fragment dropping down into the bonding combination that furnishes the bonding MO; (2) the electrostatic attraction is stronger in the heterolytic channel because of the charge separation associated with having an anion and a cation.

Supplementary Table 2. Homolytic and heterolytic activation strain and energy decomposition analyses (in kcal mol^{-1}) of *i*-$LiCF_n$ (n = 1–3), Voronoi deformation density (VDD) charges (in a.u.) of the Li fragment, and overlap integrals. [a,b]

Species	fragments	ΔE	ΔE_{strain}	ΔE_{int}	ΔE_{Pauli}	ΔV_{elstat}	ΔE_{oi}	overlap[d]	Q_{Li}[c]
***i*-LiCF₃**	Li· / ·CF_3	-66.0	52.3	-118.3	128.7	-91.6	-155.4	0.219	0.397
						(37%)	(63%)		
	Li^+ / $^-CF_3$	-152.4	3.1	-155.4	21.0	-149.4	-27.1		
						(85%)	(15%)		
***i*-LiCF₂·**	Li· / ·CF_2·	-84.3	30.0	-114.4	89.7	-68.7	-135.4	0.231	0.466
						(34%)	(66%)		
	Li^+ / $^-CF_2$·	-153.1	1.0	-154.1	16.8	-145.9	-25.0		
						(85%)	(15%)		
***i*-LiCF:**	Li· / ·CF:	-120.7	7.3	-128.0	48.4	-45.0	-131.5	0.253	0.491
						(25%)	(75%)		
	Li^+ / $^-$CF:	-155.4	0.1	-155.5	14.8	-146.2	-24.1		
						(86%)	(14%)		

[a] The values in parentheses are the percentage contributions to the total attractive interactions ($\Delta V_{elstat}+\Delta E_{oi}$). [b] For *i*-LiCF: the triplet state is lower than the singlet by 19.7 kcal/mol. The d(Li–C) = 2.135 Å. [c] VDD Li charges from spherical average-of-configuration atoms in the relaxed geometries. [d] Overlap integrals <SOMO(Li·) | SOMO(·CPh_x)> forming the Li–C pair bond. SOMO stands for Singly-Occupied Molecular Orbital.

Supplementary Table 3. Homolytic and heterolytic activation strain and energy decomposition analyses (in kcal mol^{-1}) of $LiCR_n$ interaction (R = F, Ph; n = 1–3) at the M06-2X/TZ2P level of theory. Comparison between values of the equilibrium geometry (top values) and those with the Li-C bond length (r(Li–C)) and the Li-C-X angle (α(LiCX)) as in $LiCPh_3$ geometry (1.980 Å and 83.3º, respectively, bottom values). See Supplementary Fig. 17.

$LiCF_3$	**fragments**	**Δ*E*$_{int}$**	**Δ*E*$_{Pauli}$**	**Δ*V*$_{elstat}$**	**Δ*E*$_{oi}$**	**overlap[a]**	**r(Li-C)**	**α(LiCX)**
**$LiCF_3$**	Li· / $\cdot CF_3$	-78.6	46.6	-29.7	-95.5	0.317	1.997	115.3
**$LiCF_3$ $^{(LiCPh3)}$**	Li· / $\cdot CF_3$	12.3	82.0	-46.2	-23.5	0.096	1.980	83.3
**$LiCF_2\cdot$**	Li· / $\cdot CF_2\cdot$	-94.6	35.1	-28.1	-101.6	0.322	1.997	115.3
**$LiCF_2\cdot$ $^{(LiCPh3)}$**	Li· / $\cdot CF_2\cdot$	-70.0	61.5	-45.0	-86.5	0.269	1.980	83.3
**LiCF:**	Li· / ·CF:	-123.8	21.8	-27.0	-118.6	0.330	1.997	115.3
**LiCF: $^{(LiCPh3)}$**	Li· / ·CF:	-120.7	36.3	-36.4	-120.6	0.300	1.980	83.3

i-$LiCF_3$	**fragments**	**Δ*E*$_{int}$**	**Δ*E*$_{Pauli}$**	**Δ*V*$_{elstat}$**	**Δ*E*$_{oi}$**	**overlap[a]**	**r(Li-C)**	**α(LiCX)**
***i*-$LiCF_3$**	Li· / $\cdot CF_3$	-118.3	128.7	-91.6	-155.4	0.219	2.135	58.6
***i*-$LiCF_3$ $^{(LiCPh3)}$**	Li· / $\cdot CF_3$	-7.6	80.5	-43.9	-44.1	0.088	1.980	83.3
***i*-$LiCF_2\cdot$**	Li· / $\cdot CF_2\cdot$	-114.4	89.7	-68.7	-135.4	0.231	2.135	58.6
**i-$LiCF_2\cdot$ $^{(LiCPh3)}$**	Li· / $\cdot CF_2\cdot$	-84.1	60.2	-43.5	-100.8	0.252	1.980	83.3
***i*-LiCF:**	Li· / ·CF:	-128.0	48.4	-45.0	-131.5	0.253	2.135	58.6
***i*-LiCF: $^{(LiCPh3)}$**	Li· / ·CF:	-126.2	36.3	-35.7	-126.8	0.285	1.980	83.3

$LiCPh_3$	**fragments**	**Δ*E*$_{int}$**	**Δ*E*$_{Pauli}$**	**Δ*V*$_{elstat}$**	**Δ*E*$_{oi}$**	**overlap[a]**	**r(Li-C)**	**α(LiCX)**
**$LiCPh_3$**	Li· / $\cdot CPh_3$	-50.4	157.1	-109.3	-98.2	0.071	1.980	83.3
**$LiCPh_2\cdot$**	Li· / $\cdot CPh_2\cdot$	-52.3	114.0	-83.2	-83.2	0.167	1.980	83.3
**LiCPh:**	Li· / ·CPh:	-86.4	68.7	-56.6	-98.5	0.229	1.980	83.3

[a] Overlap integrals $\langle SOMO(Li\cdot) | SOMO(\cdot CPh_x)\rangle$ forming the Li–C pair bond. SOMO stands for Singly-Occupied Molecular Orbital.

Supplementary Table 4. Energy decomposition analysis (in kcal mol^{-1}) of $LiCF_n$, *i*-$LiCF_n$ and $LiCPh_3$ (n = 1–3) at the M06-2X/TZ2P level of theory for the homolytic dissociation mode. For each species, all fragment orbitals (top values) and only occupied fragment orbitals (no virtual, bottom values) have been compared with the aim to only quantify the electron pair bond in ΔE_{oi} as the donor-acceptor interactions are prevented.

$LiCF_3$	**fragments**	ΔE_{int}	ΔE_{Pauli}	ΔV_{elstat}	ΔE_{oi}
**$LiCF_3$**	Li· / ·CF_3	-78.6	46.6	-29.7	-95.5
**$LiCF_3$ (no virtual)**	Li· / ·CF_3	-51.1	46.6	-29.7	-68.0
**$LiCF_2$·**	Li· / ·CF_2·	-94.6	35.1	-28.1	-101.6
**$LiCF_2$· (no virtual)**	Li· / ·CF_2·	-64.9	35.1	-28.1	-71.9
**LiCF:**	Li· / ·CF:	-123.8	21.8	-27.0	-118.6
**LiCF: (no virtual)**	Li· / ·CF:	-77.8	21.8	-27.0	-72.6

i-$LiCF_3$	**fragments**	ΔE_{int}	ΔE_{Pauli}	ΔV_{elstat}	ΔE_{oi}
***i*-$LiCF_3$**	Li· / ·CF_3	-118.3	128.7	-91.6	-155.4
***i*-$LiCF_3$ (no virtual)**	Li· / ·CF_3	-59.3	128.7	-91.6	-96.5
***i*-$LiCF_2$·**	Li· / ·CF_2·	-114.4	89.7	-68.7	-135.4
**i-$LiCF_2$· (no virtual)**	Li· / ·CF_2·	-58.9	89.7	-68.7	-80.0
***i*-LiCF:**	Li· / ·CF:	-128.0	48.4	-45.0	-131.5
***i*-LiCF: (no virtual)**	Li· / ·CF:	-68.9	48.4	-45.0	-72.3

$LiCPh_3$	**fragments**	ΔE_{int}	ΔE_{Pauli}	ΔV_{elstat}	ΔE_{oi}
**$LiCPh_3$**	Li· / ·CPh_3	-50.4	157.1	-109.3	-98.2
**$LiCPh_3$ (no virtual)**	Li· / ·CPh_3	-9.1	157.1	-109.3	-56.8
**$LiCPh_2$·**	Li· / ·CPh_2·	-52.3	114.0	-83.2	-83.2
**$LiCPh_2$·(no virtual)**	Li· / ·CPh_2·	-7.9	114.0	-83.2	-38.7
**LiCPh:**	Li· / ·CPh:	-86.4	68.7	-56.6	-98.5
**LiCPh: (no virtual)**	Li· / ·CPh:	-40.9	68.7	-56.6	-53.0

Supplementary Table 5. Cartesian coordinates and ADF absolute energies (in kcal mol^{-1}) of the systems under analysis computed at the M06-2X/TZ2P level of theory.

$LiCF_3$ (-856.5)

1	C	-0.00000000	-0.00000000	-0.04577359
2	F	-0.62399719	1.08079482	-0.63530448
3	F	-0.62399719	-1.08079482	-0.63530448
4	F	1.24799438	0.00000000	-0.63530448
5	Li	0.00000000	0.00000000	1.95168702

$LiCPh_3$ (-6997.1)

1	C	-0.00000000	-0.00000000	-0.47448398
2	H	-2.44564134	0.63458077	-1.44137847
3	H	0.67325760	-2.43527791	-1.44137847
4	H	1.77238374	1.80069714	-1.44137847
5	Li	-0.00000000	0.00000000	1.50548285
6	C	1.44990765	0.00593062	-0.30394377
7	C	2.12699736	-0.95581637	0.48181663
8	C	3.50007548	-0.88797551	0.70634809
9	C	4.25241808	0.14009293	0.17148015
10	C	3.60719073	1.10884877	-0.60110542
11	C	2.25072595	1.04657434	-0.83041793
12	H	1.58613240	-1.81083120	0.87399759
13	H	3.97521920	-1.65652558	1.30210041
14	H	5.31900766	0.18969160	0.33773806
15	H	4.18166914	1.91413419	-1.04030163
16	C	-0.71981776	-1.25862217	-0.30394377
17	C	-1.89125993	-1.36412557	0.48181663
18	C	-2.51904709	-2.58716653	0.70634809
19	C	-2.00488500	-3.75274855	0.17148015
20	C	-0.84330417	-3.67834319	-0.60110542
21	C	-0.21900301	-2.47247302	-0.83041793
22	H	-2.36129202	-0.46821534	0.87399759
23	H	-3.42220283	-2.61437802	1.30210041
24	H	-2.49522608	-4.70124156	0.33773806
25	H	-0.43314574	-4.57849880	-1.04030163
26	C	-0.73008989	1.25269155	-0.30394377
27	C	-0.23573742	2.31994193	0.48181663
28	C	-0.98102839	3.47514205	0.70634809
29	C	-2.24753308	3.61265561	0.17148015
30	C	-2.76388656	2.56949442	-0.60110542
31	C	-2.03172294	1.42589868	-0.83041793
32	H	0.77515963	2.27904655	0.87399759
33	H	-0.55301637	4.27090360	1.30210041
34	H	-2.82378158	4.51154995	0.33773806
35	H	-3.74852341	2.66436461	-1.04030163

***i*-$LiCF_3$ (**-858.9)

1	C	-0.00004166	-0.00000034	0.08402165
2	F	-0.62215437	1.07763105	-0.67574670

3	F	-0.62215399	-1.07762971	-0.67574818
4	F	1.24435014	0.00000033	-0.67577414
5	Li	0.00000416	0.00000179	-2.05121326

SUPPLEMENTARY FIGURES

Supplementary Fig. 1: Main bond lengths (in Å) and angles (in degrees) of $LiCF_3$, *i*-$LiCF_3$ and $LiCPh_3$.

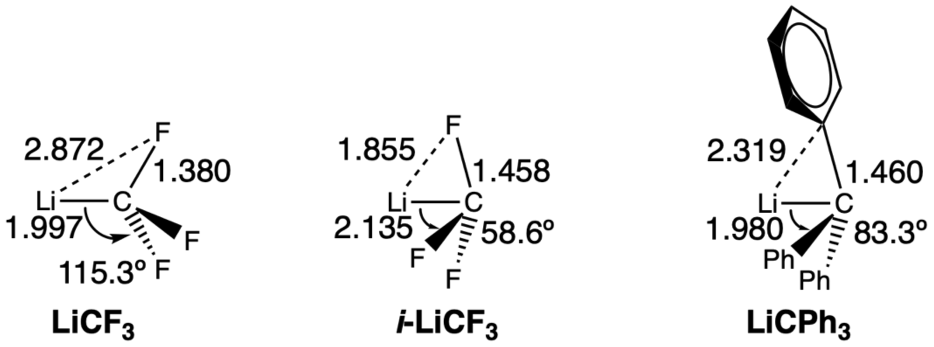

Supplementary Fig. 2: Orbital interaction diagram for $LiCF_3$ (top value) and *i*-$LiCF_3$ (bottom value in italics). Only the Singly-Occupied Molecular Orbitals (SOMOs) forming the Li-C bond are included. Energies of the SOMOs (values below energy levels, in eV), Gross Mulliken contributions (values next to dashed lines), Gross Mulliken populations (pop., in electrons), and their overlaps <SOMO(Li·) | SOMO(·CF_3)> forming the Li–C pair bond are also included.

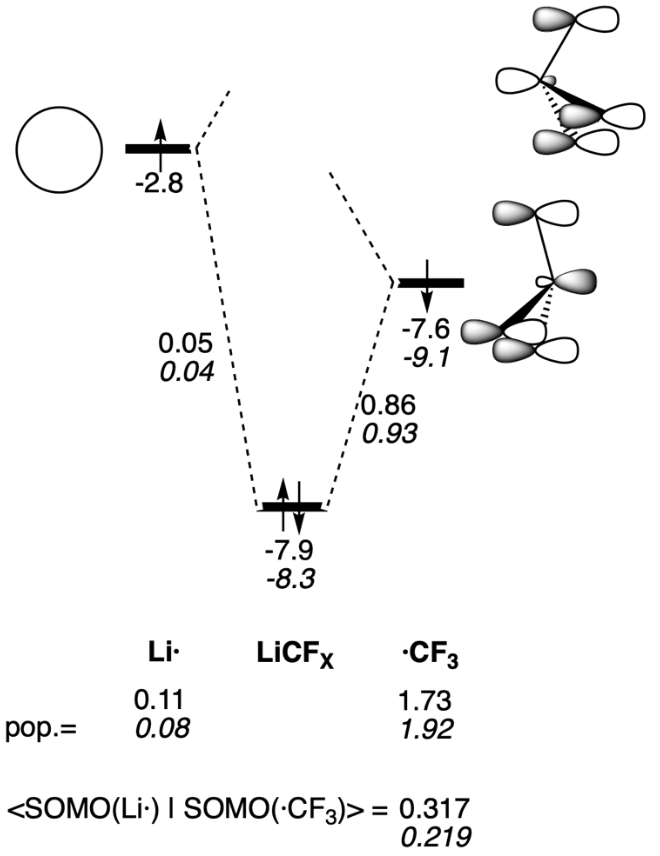

Supplementary Fig. 3: Singly-Occupied Molecular Orbitals (SOMOs) of Li and CF_3 in $LiCF_3$. Energies (in eV) and overlaps <SOMO(Li·) | SOMO(·CF_x)> forming the Li–C bond are also included for $LiCF_3$ (left value) and *i*-$LiCF_3$ (right value).

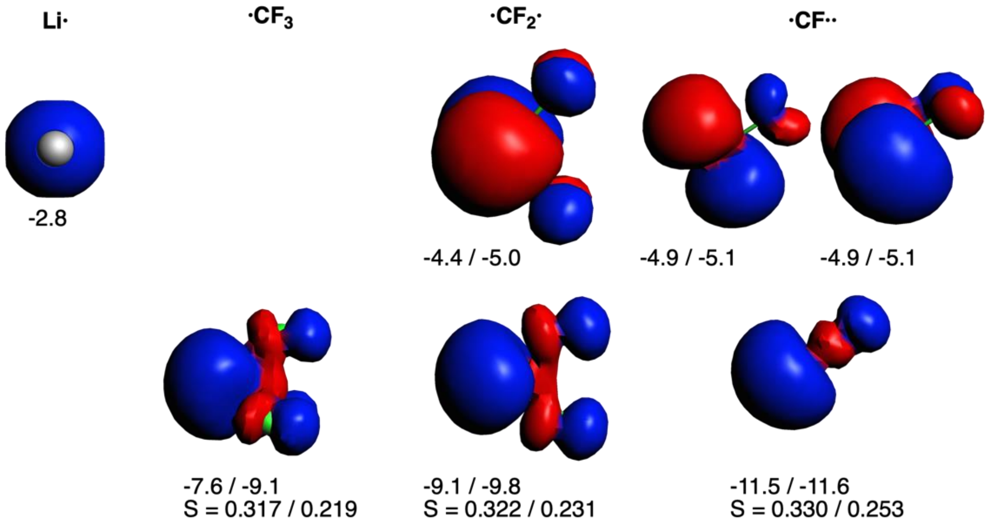

Supplementary Fig. 4: Orbital interaction diagram for $LiCF_3$ involving the interaction between the Singly-Occupied Molecular Orbitals (SOMOs) forming the Li–C bond. Higher occupied molecular orbitals for $LiCF_3$ are depicted in the center. Energies of the SOMOs (values below energy levels, in eV), Gross Mulliken contributions (values next to dashed lines) are included.[a]

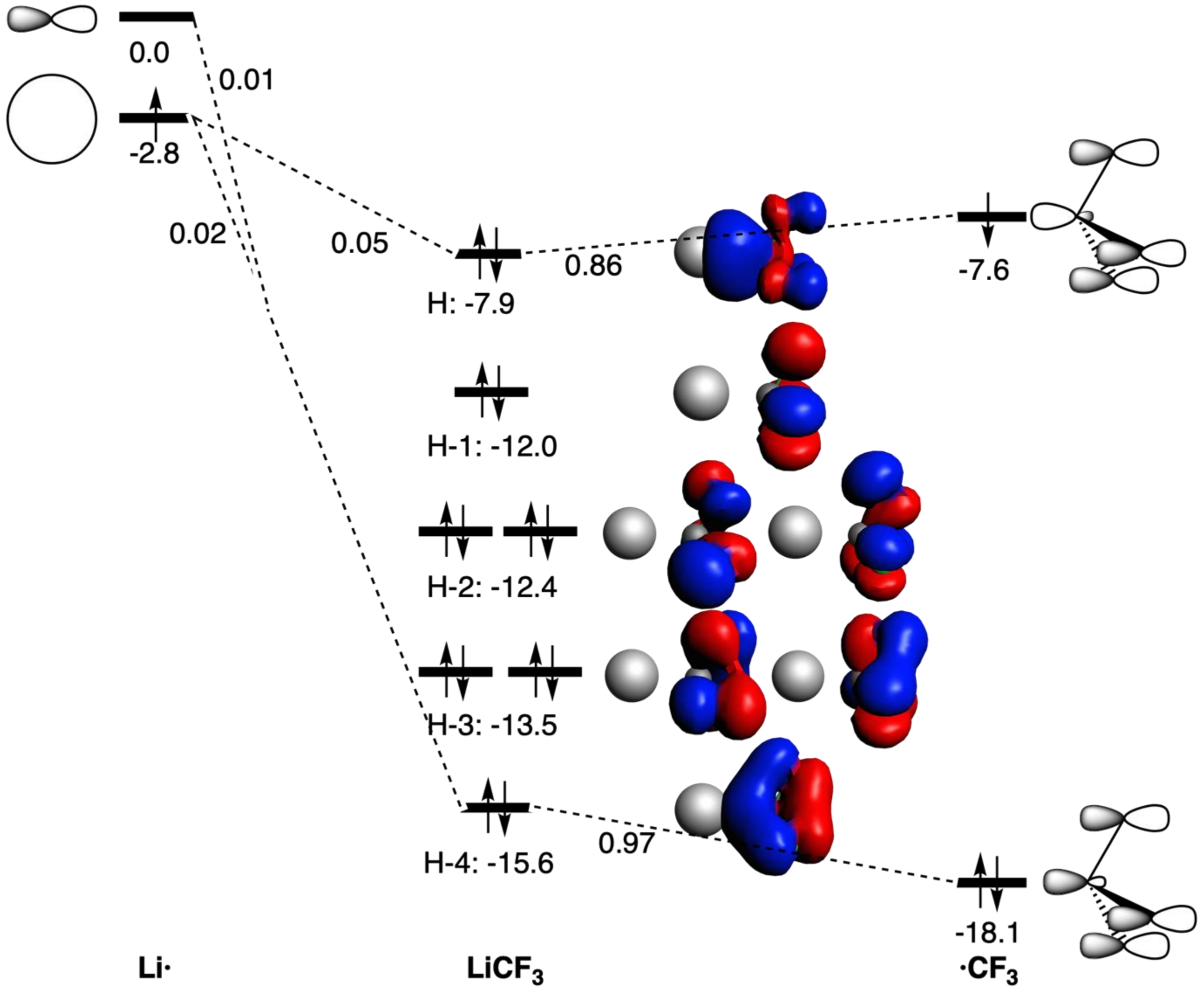

[a] Gross Mulliken populations for the Lowest Unoccupied Molecular Orbital (LUMO) of Li· are 0.09, 0.11 and 0.11 e. for $LiCF_3$, $LiCF_2$·, and LiCF··, respectively, 0.03, 0.03 and 0.04 for for *i*-$LiCF_3$, *i*-$LiCF_2$·, and *i*-LiCF··, respectively, and 0.06, 0.06 and 0.05 for $LiCPh_3$, $LiCPh_2$·, and LiCPh··, respectively. These values correspond to the small charge transfer between the bonding p_z fragment molecular orbital of $·CF_x$ and the p_z empty orbital of Li·.

Supplementary Fig. 5: Orbital interaction diagram for $LiCPh_3$. Only the Singly-Occupied Molecular Orbitals (SOMOs) forming the C–Li bond are included. Energies of the SOMOs (values below energy levels, in eV), Gross Mulliken contributions (values next to dashed lines), Gross Mulliken populations (pop., in electrons), and its overlap <SOMO(Li·) | SOMO(·CPh_3)> forming the C-Li pair bond are also included.

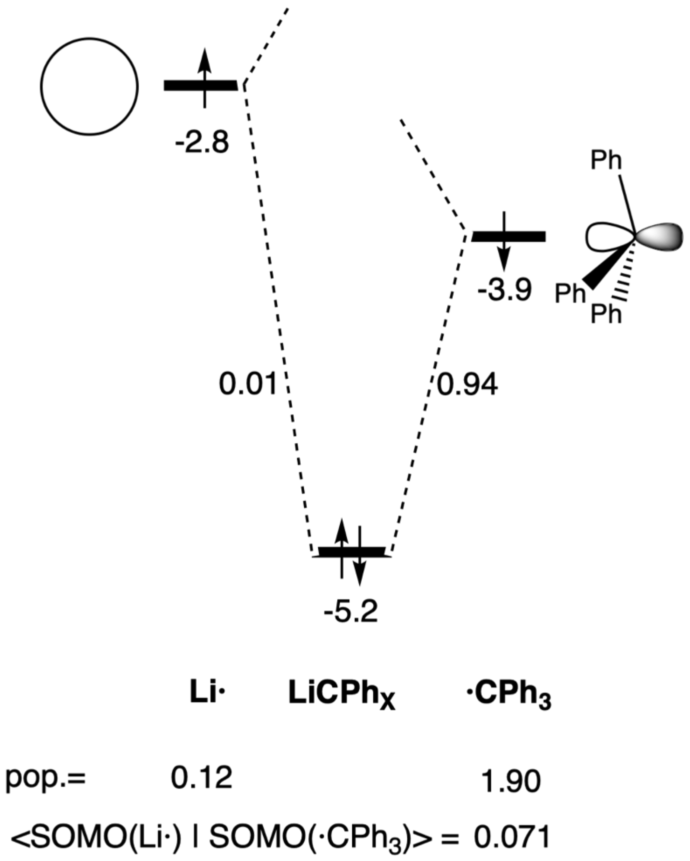

Supplementary Fig. 6: Singly-Occupied Molecular Orbitals (SOMOs) of $LiCPh_3$, together with their CPh_2 and CPh radical species (at the same geometry adopted for CPh_3). Energies (in eV) and overlaps <SOMO(Li·) | SOMO(·CPh_x)> forming the C-Li bond are also included.

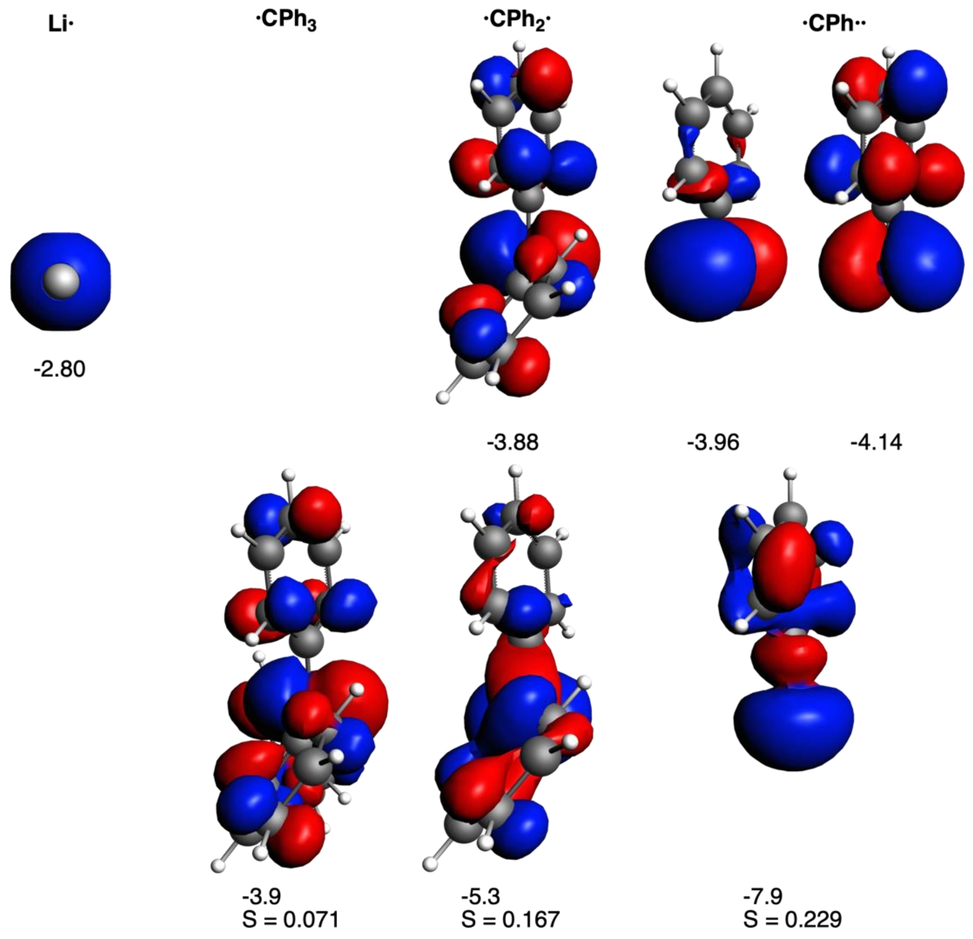

Supplementary Fig. 7: Highest Occupied Molecular Orbitals (HOMO, bottom) and Lowest Unoccupied Molecular Orbitals (LUMO, top) of $LiCF_3$, *i*-$LiCF_3$ and $LiCPh_3$. Red and blue isosurfaces represent electron and hole distributions of occupied orbitals, whereas orange and turquoise are of virtual orbitals.

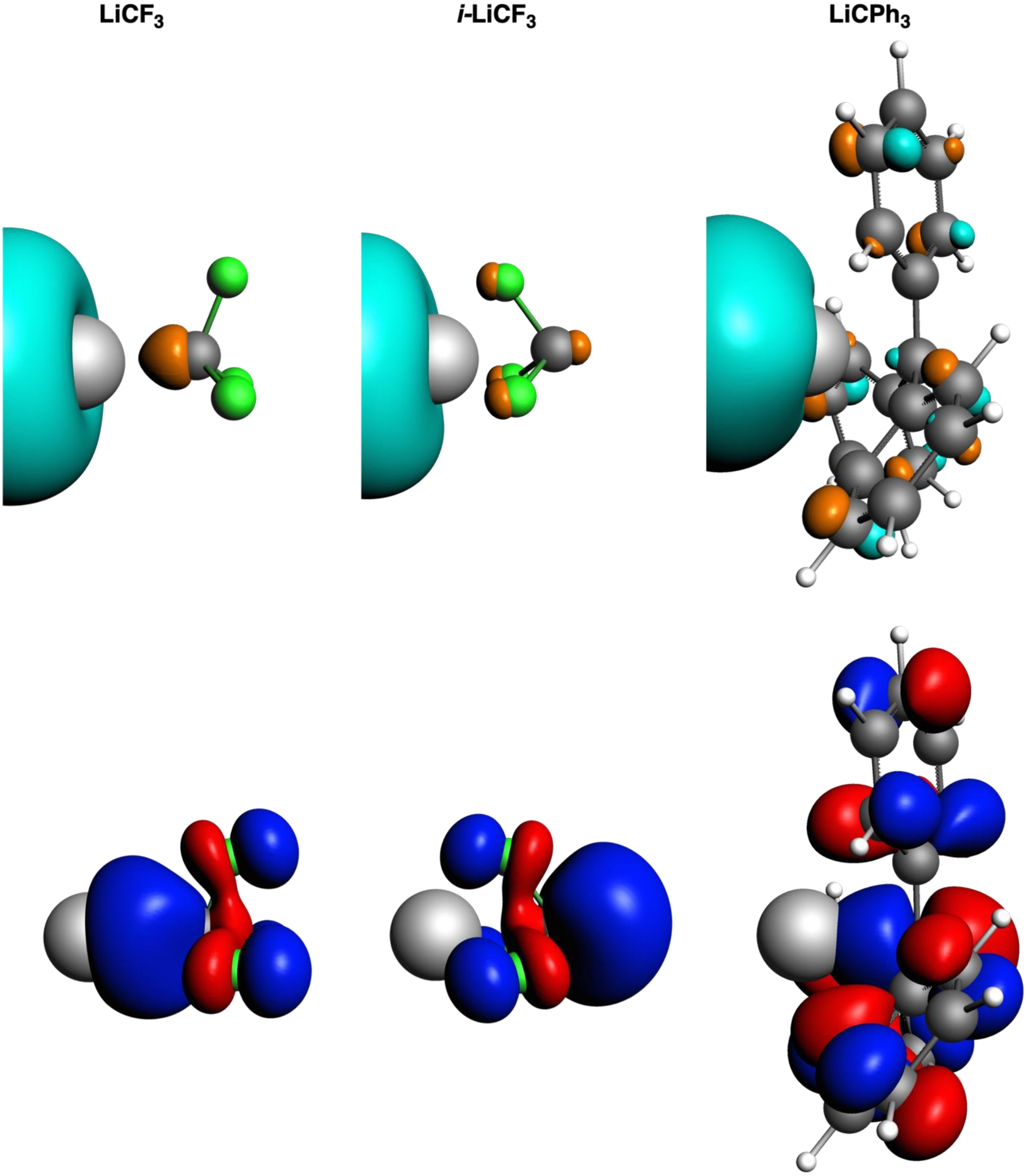

Supplementary Fig. 8: Schematic Non-Bonding Molecular Orbitals (NBMO) obtained by Hückel theory for uneven alternating π systems (left) and SOMO of CPh_3 (right).

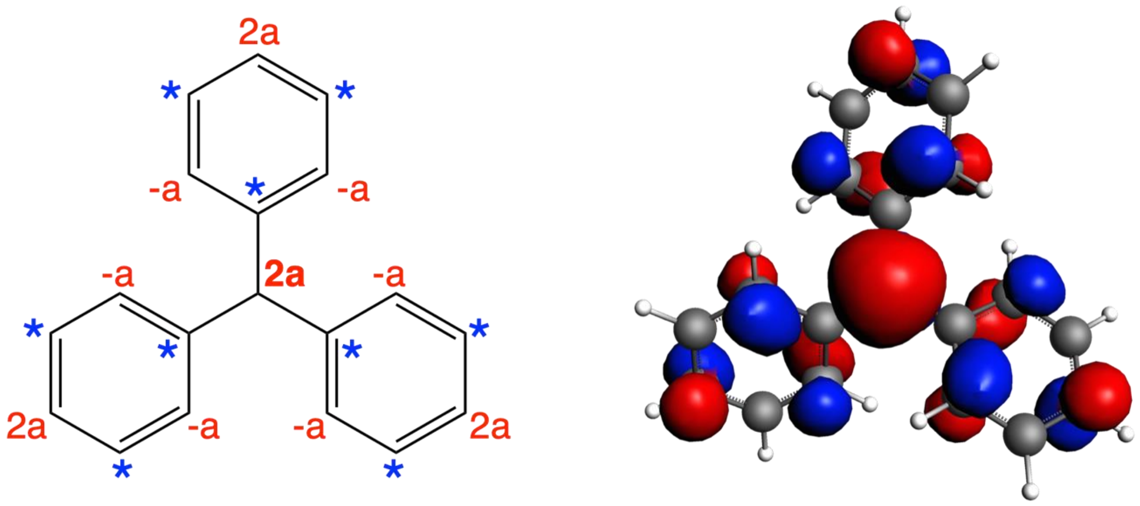

Supplementary Fig. 9: Overlap density between the two Singly-Occupied Molecular Orbitals (SOMOs) of $LiCF_3$, *i*-$LiCF_3$, and $LiCPh_3$. Isosurfaces of the superposed SOMO of Li· and the SOMO of $\cdot CR_3$ (R = F, Ph) to construct $LiCF_3$, *i*-$LiCF_3$, and $LiCPh_3$ (left, isovalue = 0.05 a.u.). Red and blue isosurfaces represent electron and hole distributions. Overlap density between the two SOMOs (right, isovalue = 0.001 a.u.). Purple and orange isosurfaces indicate in-phase and out-of-phase overlap, respectively.

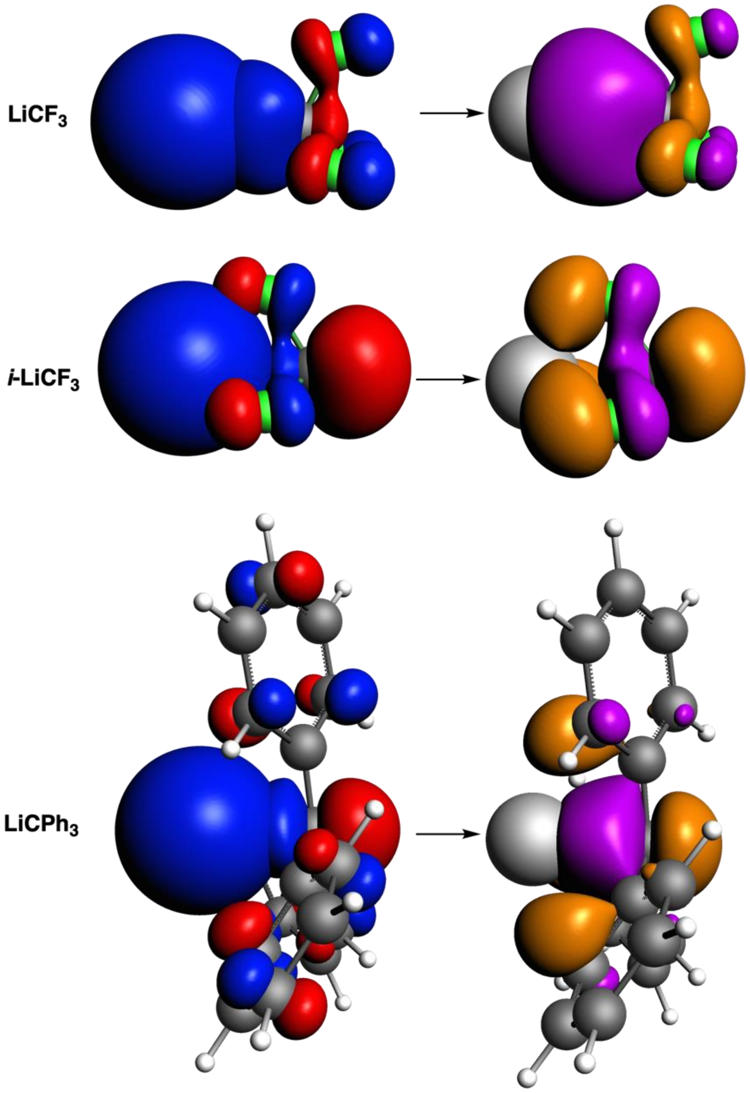

Supplementary Fig. 10: Overlap density between the two Singly-Occupied Molecular Orbitals (SOMOs) of $LiCF_3$. Isosurfaces of the SOMO of Li· and the SOMO of $\cdot CF_3$ to construct $LiCF_3$ and its radical species (left, isovalue = 0.05 a.u.). Red and blue isosurfaces represent electron and hole distributions. Overlap density between the two SOMOs (right, isovalue = 0.001 a.u.). Purple and orange isosurfaces indicate in-phase and out-of-phase overlap, respectively.

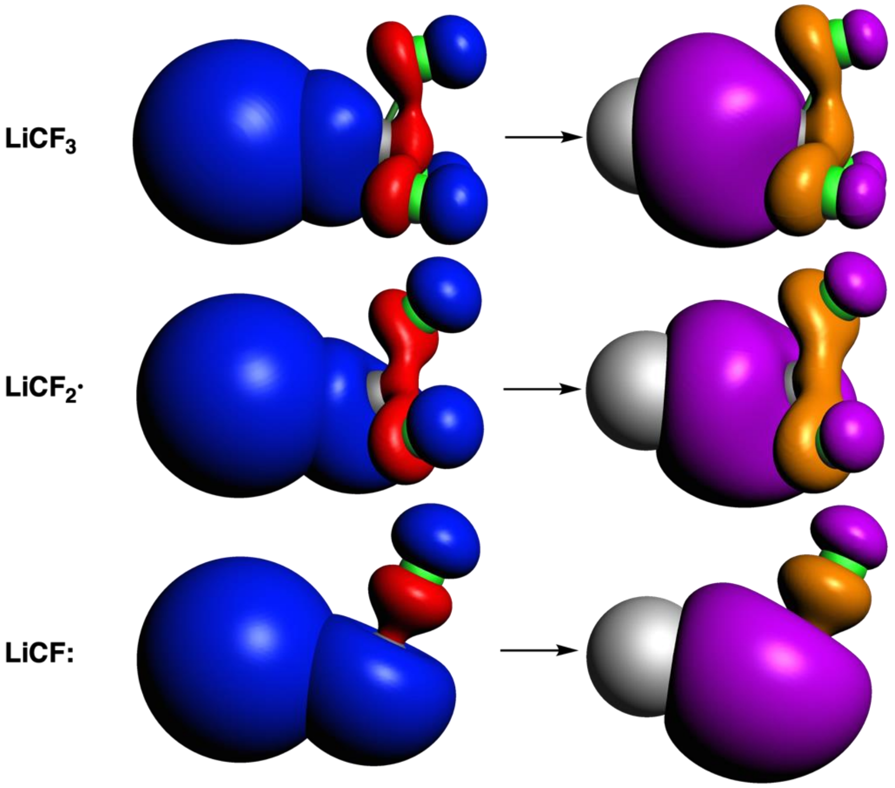

Supplementary Fig. 11: Overlap density between the two Singly-Occupied Molecular Orbitals (SOMOs) of *i*-LiCF$_3$. Isosurfaces of the SOMO of Li· and the SOMO of ·CF$_3$ to construct *i*-LiCF$_3$ and its radical species (left, isovalue = 0.05 a.u.). Red and blue isosurfaces represent electron and hole distributions. Overlap density between the two SOMOs (right, isovalue = 0.001 a.u.). Purple and orange isosurfaces indicate in-phase and out-of-phase overlap, respectively.

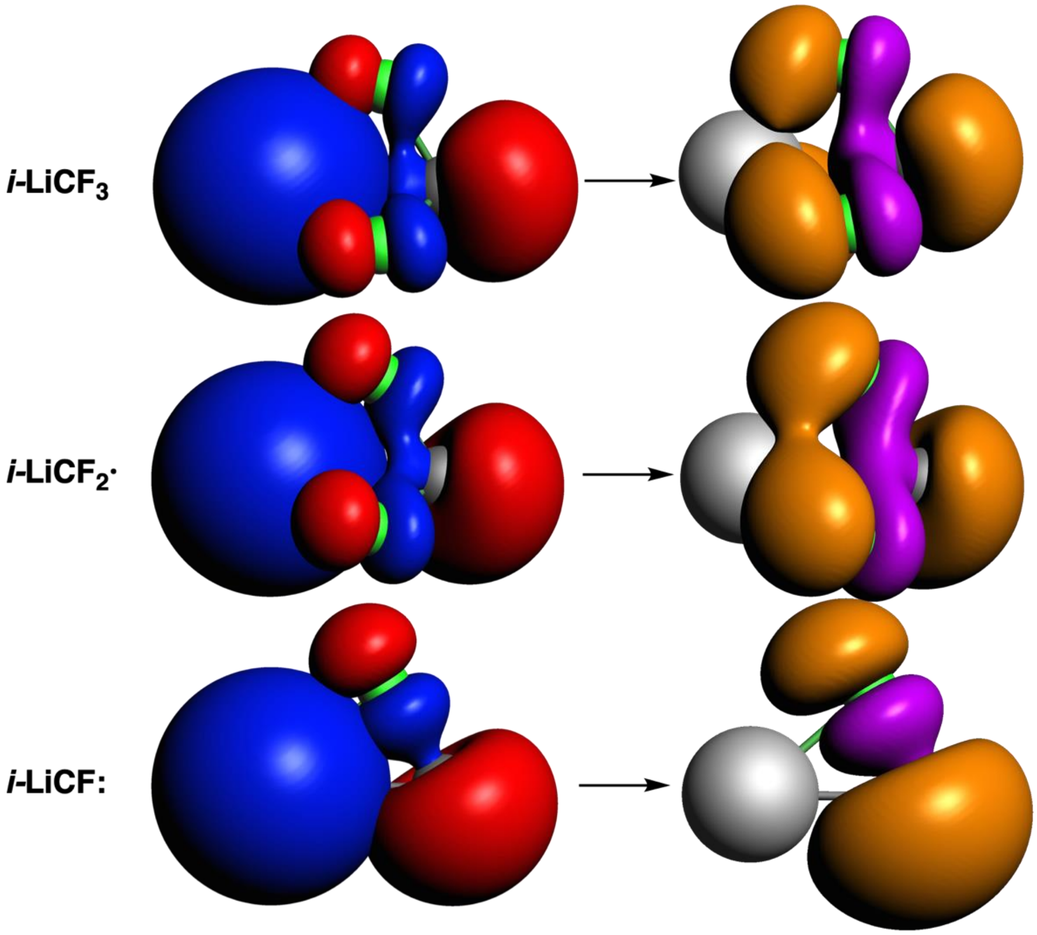

Supplementary Fig. 12: Overlap density between the two Singly-Occupied Molecular Orbitals (SOMOs) of $LiCPh_3$. Isosurfaces of the SOMO of Li· and the SOMO of $\cdot CPh_3$ to construct $LiCPh_3$ and its radical species (left, isovalue = 0.05 a.u.). Red and blue isosurfaces represent electron and hole distributions. Overlap density between the two SOMOs (right, isovalue = 0.001 a.u.). Purple and orange isosurfaces indicate in-phase and out-of-phase overlap, respectively.

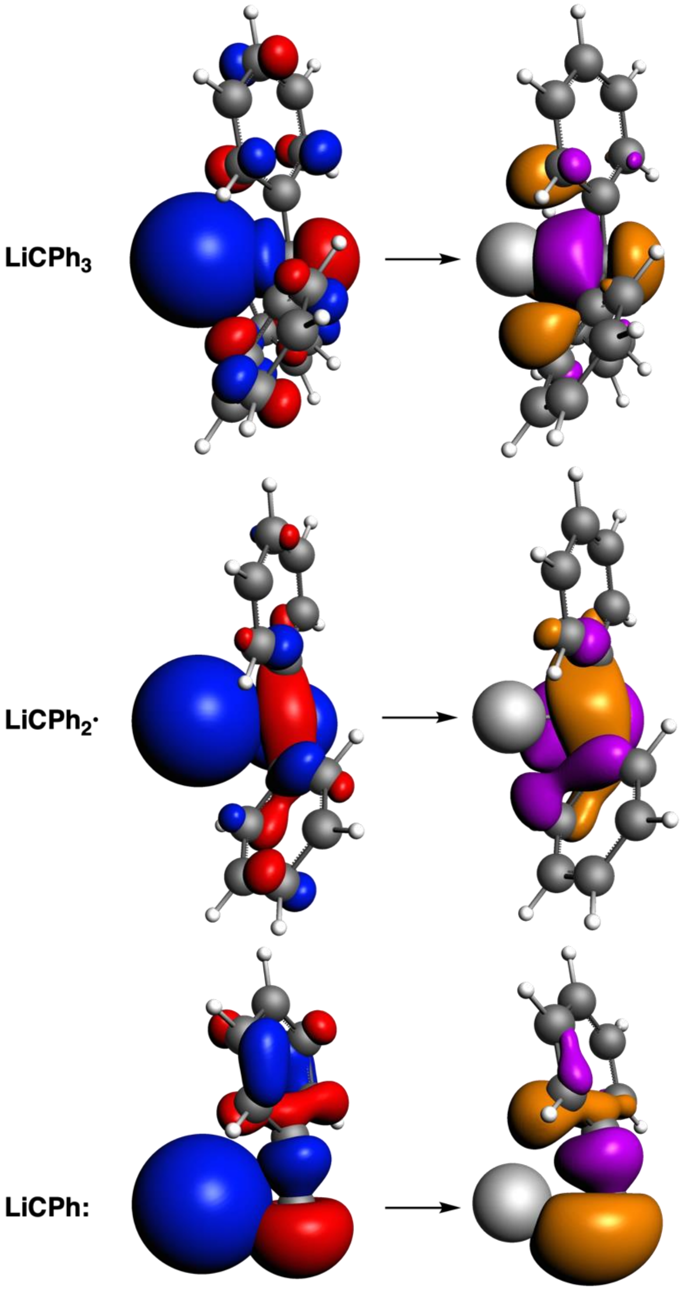

Supplementary Fig. 13: Energy Decomposition Analysis (EDA) scans. EDA scan (in kcal mol^{-1}) of a) $LiCF_3$, b) *i*-$LiCF_3$, and c) $LiCPh_3$, as a function of the Li-C bond length (in Å). CX_3 (solid), CX_2 (round dot) and CX (dash).

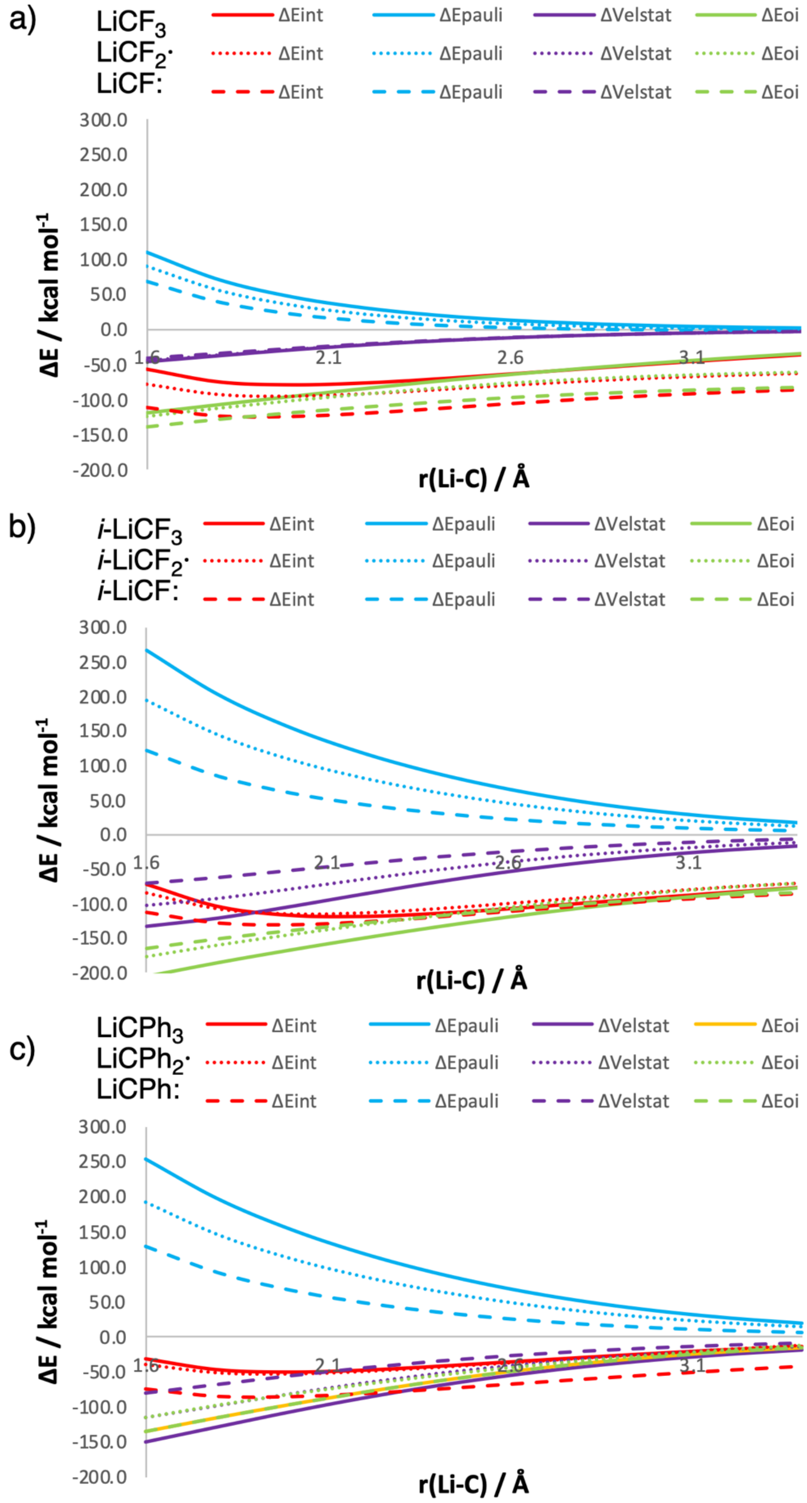

Supplementary Fig. 14: Energy Decomposition Analysis (EDA) scans comparisons. EDA scan (in kcal mol^{-1}) of a) $LiCF_3$ (solid) vs. *i*-$LiCF_3$ (round dot), and b) $LiCF_3$ (solid) vs. $LiCPh_3$ (round dot) as a function of the Li-C bond length (in Å).

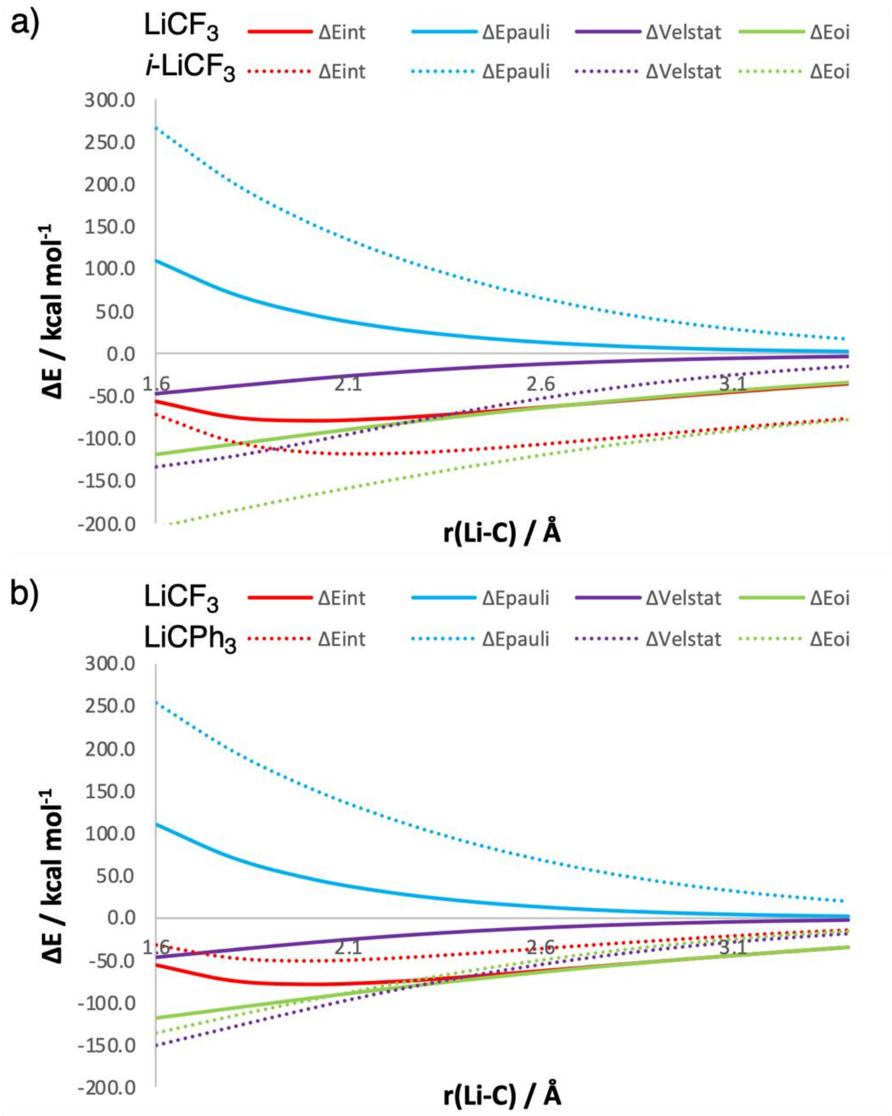

Supplementary Fig. 15: Voronoi Deformation Density (VDD) charge analyses. VDD charges (in a.u.) of the two fragments in $LiCF_3$, *i*-$LiCF_3$ and $LiCPh_3$ in the EDA analysis: homolytic (top value) and heterolytic (bottom value in italics).

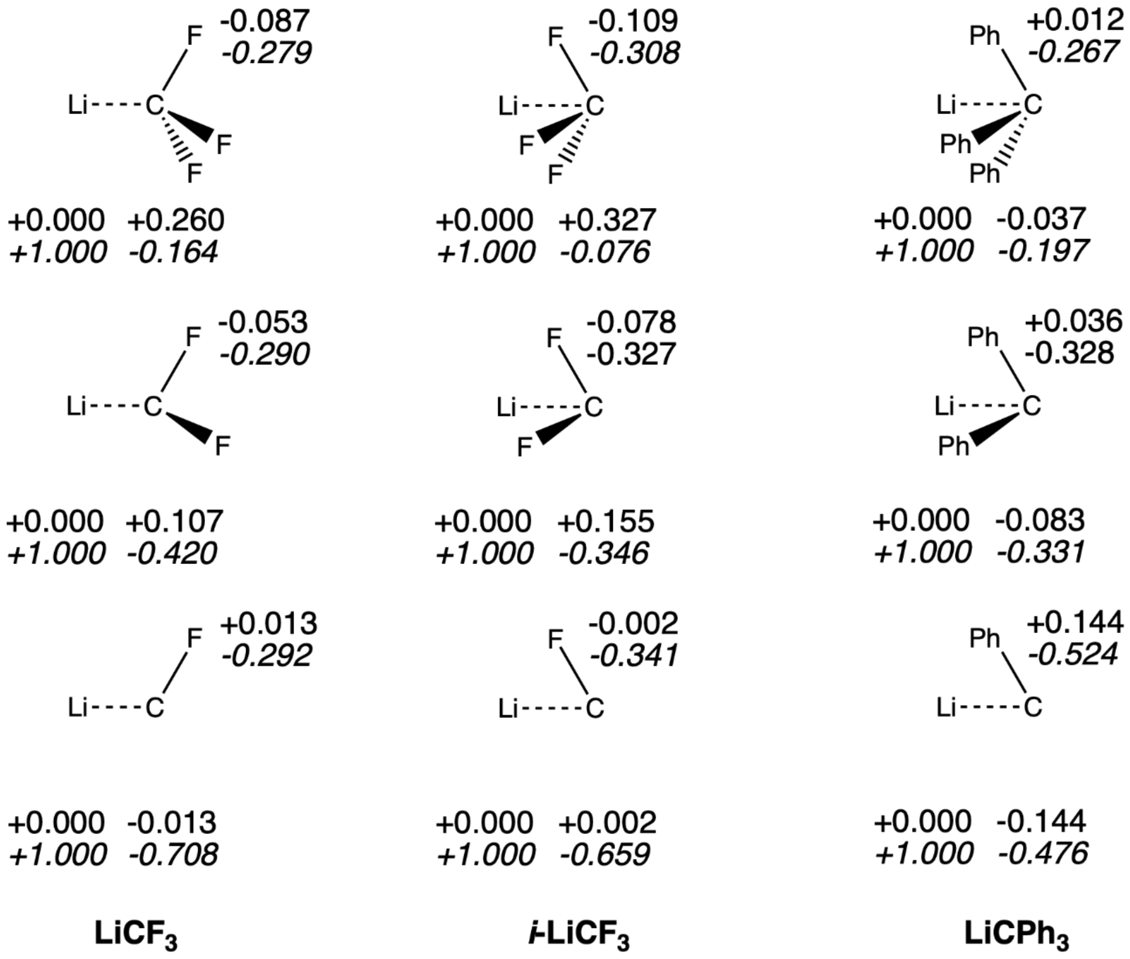

Supplementary Fig. 16: Overlaps <SOMO(Li·) | SOMO(·CF_x)> forming the C-Li pair of $LiCF_3$, *i*-$LiCF_3$, and $LiCPh_3$, as a function of the Li-C bond length (in Å). CX_3 (solid), CX_2 (round dot) and CX (dash). SOMO stands for Singly-Occupied Molecular Orbital.

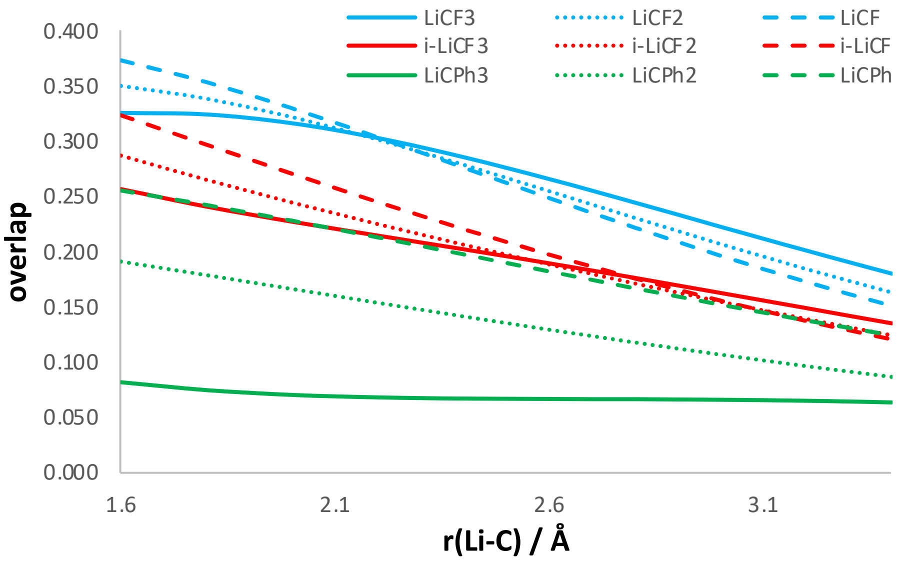

Supplementary Fig. 17: Main bond lengths (in Å) and angles (in degrees) of $LiCF_3$, *i*-$LiCF_3$ and $LiCPh_3$ and comparison to their model systems adapted to $LiCPh_3$ geometry.

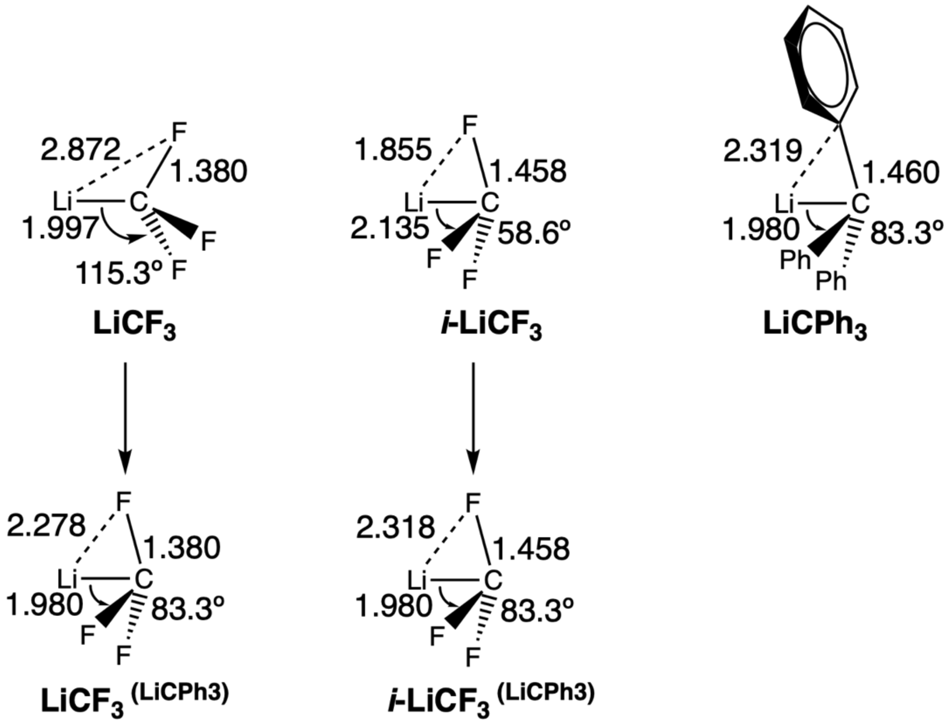

Supplementary Fig. 18: Plot of the Extended Transition State – Natural Orbitals for Chemical Valence (ETS-NOCV) deformation densities of $LiCF_3$, *i*-$LiCF_3$ and $LiCPh_3$. The direction of charge flow is red→blue (isovalue = 0.003). The eigenvalues |ν| indicate the relative size of the charge flow, whereas orbital interactions are in kcal mol^{-1}. Homolytic breaking has been considered (alpha spin on Li·).

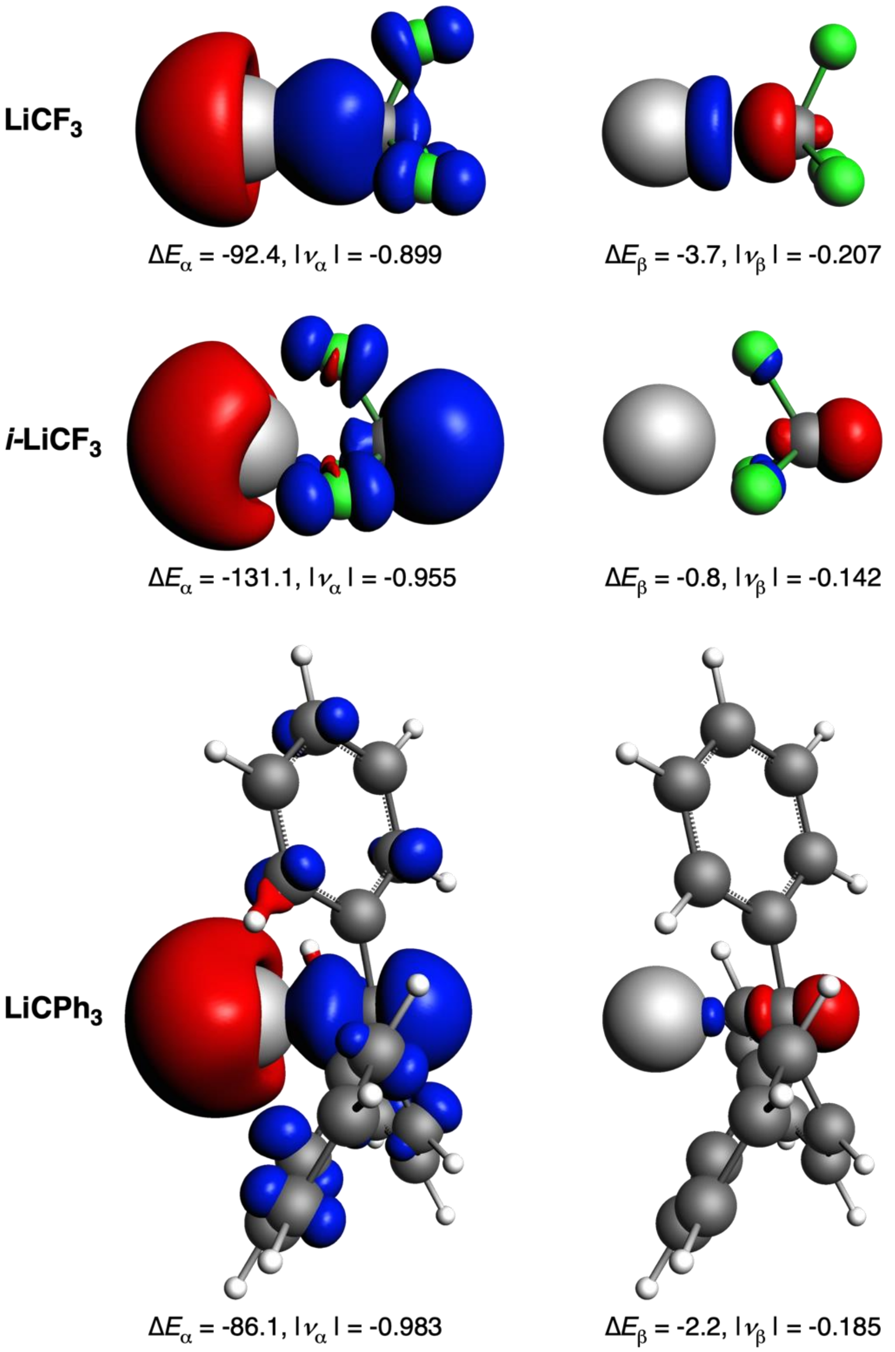

Supplement: Supplementary file 1 — Supplementary Information [file 41467_2023_39498_MOESM1_ESM.pdf]
